# Thrombocytopenia With Absent Radii Syndrome With an Unusual Urological Pathology: A Case Report

**DOI:** 10.7759/cureus.23991

**Published:** 2022-04-09

**Authors:** Rebecca Farlett, Aarti Kulkarni, Bettina Thomas, Janardhan Mydam

**Affiliations:** 1 Neonatology, John H. Stroger, Jr. Hospital of Cook County, Chicago, USA; 2 Neonatal Intensive Care Unit, John H. Stroger, Jr. Hospital of Cook County, Chicago, USA; 3 Pediatrics, John H. Stroger, Jr. Hospital of Cook County, Chicago, USA

**Keywords:** case report, rare condition, pediatrics, rare genetic diseases, cow's milk protein allergy, vesicoureteral reflux, thrombocytopenia with absent radii (tar) syndrome

## Abstract

Thrombocytopenia with absent radii (TAR) syndrome is a rare congenital syndrome that follows an autosomal recessive pattern of inheritance. TAR syndrome is characterized by thrombocytopenia and bilateral absence (aplasia) of the radii of the forearms. This syndrome can be associated with defects within the skeletal, cardiac, renal, or gastrointestinal systems. It is important for clinicians treating patients with TAR syndrome to be aware of the myriad of complications that may arise in the other organ systems in order to promptly diagnose and treat any associated anomalies. We present a case of an African American infant diagnosed with TAR syndrome who was also found to have grade 5 vesicoureteral reflux and moderate right hydronephrosis, as well as cow’s milk protein allergy.

## Introduction

Thrombocytopenia with absent radii (TAR) syndrome is a very rare congenital condition inherited in an autosomal recessive pattern [1]. The first substantial investigation of the syndrome, which provided the syndrome its name, was published in 1969 [2]. Since then, the incidence has been reported to be between 0.42 per 100,000 live births and 1:200,000-1:100,000 [3,4]. The diagnosis may be confirmed if a patient has a microdeletion on chromosome 1q21 resulting in a null allele of the RNA-binding motif protein 8A (RBM8A) gene and a hypomorphic allele of the other RBM8A gene due to a pathogenic variant single nucleotide polymorphism (SNP) [4]. The syndrome is characterized by a bilateral absence of radii accompanied by the presence of both thumbs and hypomegakaryocytic thrombocytopenia (HMT) [5]. Upon diagnosis of TAR syndrome, the thrombocytopenia can be quite severe; approximately 95% of patients with the condition have a platelet count of <50,000/uL, normalizing over time in most patients [4]. Immediately after birth, approximately 50% of infants with TAR syndrome have symptomatic thrombocytopenia, and by four months of life, 90% will be symptomatic [6].

TAR syndrome has also been found to increase the occurrence of anomalies within hematologic, cardiovascular [4], gastrointestinal, and skeletal systems [7]. Cardiovascular anomalies may include tetralogy of Fallot or atrial septal defects [4]. Skeletal anomalies may result in a higher risk of dental and craniofacial trauma in this patient population [7]. It has been reported that more than 60% of those with TAR syndrome have accompanying cow's milk protein allergy (CMPA) [8]. The impact of the syndrome on the health of an individual is profound. Both morbidity and mortality are increased among those with TAR syndrome, with the leading causes of death being intracranial hemorrhage and cardiovascular events [4].

Within the current case report, we detail the signs, symptoms, diagnosis, and treatment regimens of an infant with TAR syndrome. We further explore the complications related to the syndrome experienced by our patient, including CMPA, renal abnormalities, and grade 5 vesicoureteral reflux (VUR). Although genitourinary and renal anomalies have been associated with TAR syndrome, to our knowledge, this is the first case of VUR in a patient with TAR syndrome.

## Case presentation

**Figure 1:**
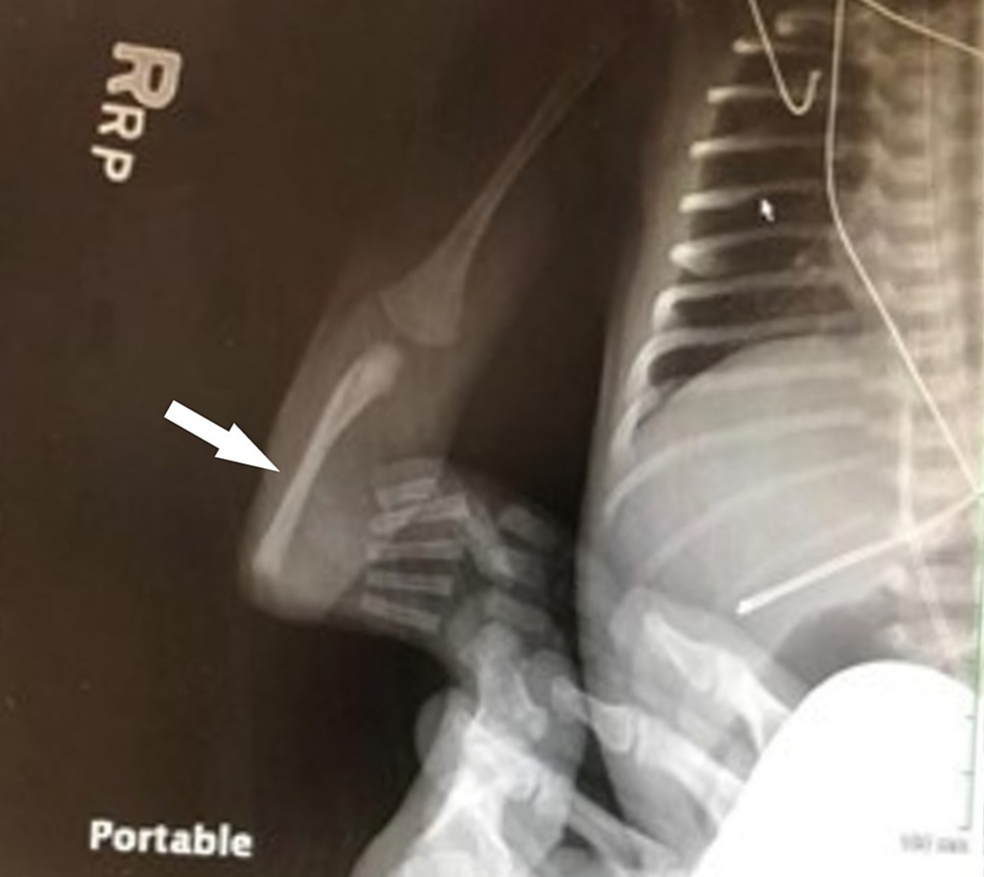
X-ray of the right arm demonstrating absent radius

**Figure 2:**
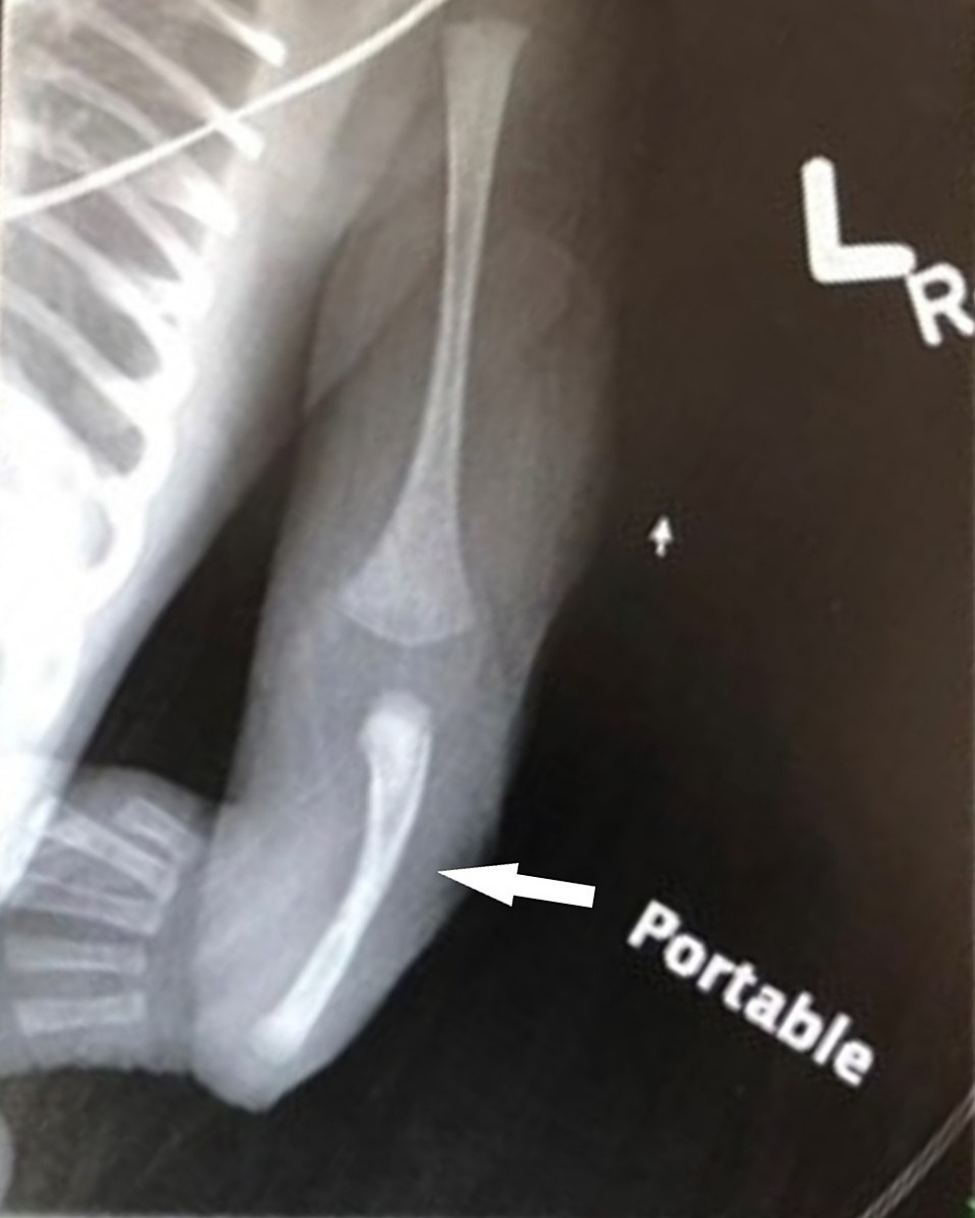
X-ray of the left arm demonstrating absent radius

An African American male was born to a 26-year-old gravida 4 para 3 mother. The mother had a past medical history significant for obesity, anemia, depression, coronavirus disease 2019 (COVID-19), and pyelonephritis in her third trimester. Prenatal medications included escitalopram, iron, and prenatal vitamins. Her prenatal infectious laboratory results were negative other than vaginal swab positive for group B *Streptococcus* that was adequately treated with penicillin. The infant was born via cesarean section, at an outside hospital, at 37 weeks due to late decelerations and failure to progress. Rupture of membranes occurred three hours prior to delivery with clear fluid. The Apgar scores were six and eight at one minute and five minutes, respectively. The infant did not have a strong cry at birth and required one minute of positive pressure ventilation (PPV) followed by continuous positive airway pressure (CPAP). He was subsequently transferred to the newborn nursery on CPAP. Initial exam revealed both arms were hyperflexed at the elbow with radially deviated hands and bilateral presence of the thumbs. An X-ray of the upper limbs revealed absent radii bilaterally (Figures 1, 2). Initial complete blood count (CBC) showed thrombocytopenia with a platelet count of 53,000/uL. At this stage, the patient was transferred to our hospital for further multi-disciplinary management.

The patient was also found to be in respiratory distress, believed to be most likely secondary to transient tachypnea of the newborn (TTN), and the patient was weaned to room air by 18 hours of life (HOL). The patient completed 36 hours of empiric antibiotics to rule-out sepsis; a blood culture report produced a negative result. He was also on phototherapy for three days for hyperbilirubinemia, which resolved following treatment.

Due to suspected TAR syndrome, hematology, orthopedics, urology, gastroenterology (GI), and genetics were consulted. The patient was started on formula with Nutramigen, as per the recommendation of hematology and GI. Genetic testing for TAR syndrome was sent and confirmed the diagnosis. His microarray results demonstrated that "the copy number loss of 1q21.1 involves several protein-coding genes, including RBM8A." Recurrent reciprocal deletions and duplications of 1q21.1 partially overlapping this interval have been associated with variable phenotypes, including dysmorphic features, developmental delay, intellectual disability, autistic features, skeletal malformations, cardiac defects, and genitourinary abnormalities. Additionally, this interval fully encompasses the region associated with autosomal recessive TAR syndrome.

**Figure 3:**
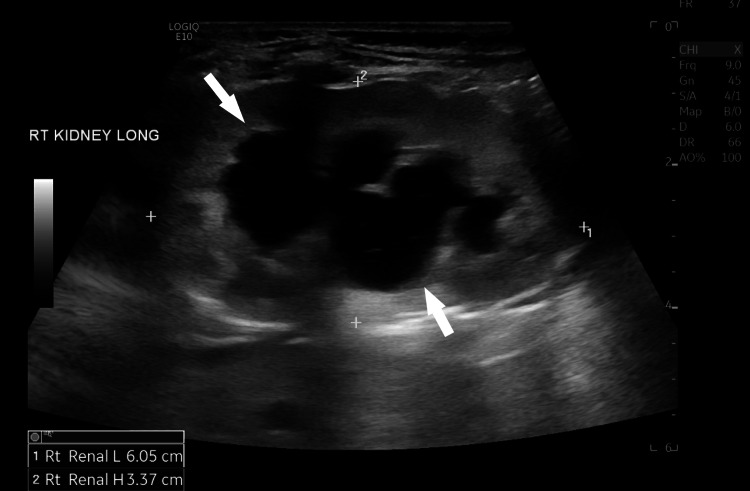
Renal ultrasound showing moderate right hydronephrosis

**Figure 4:**
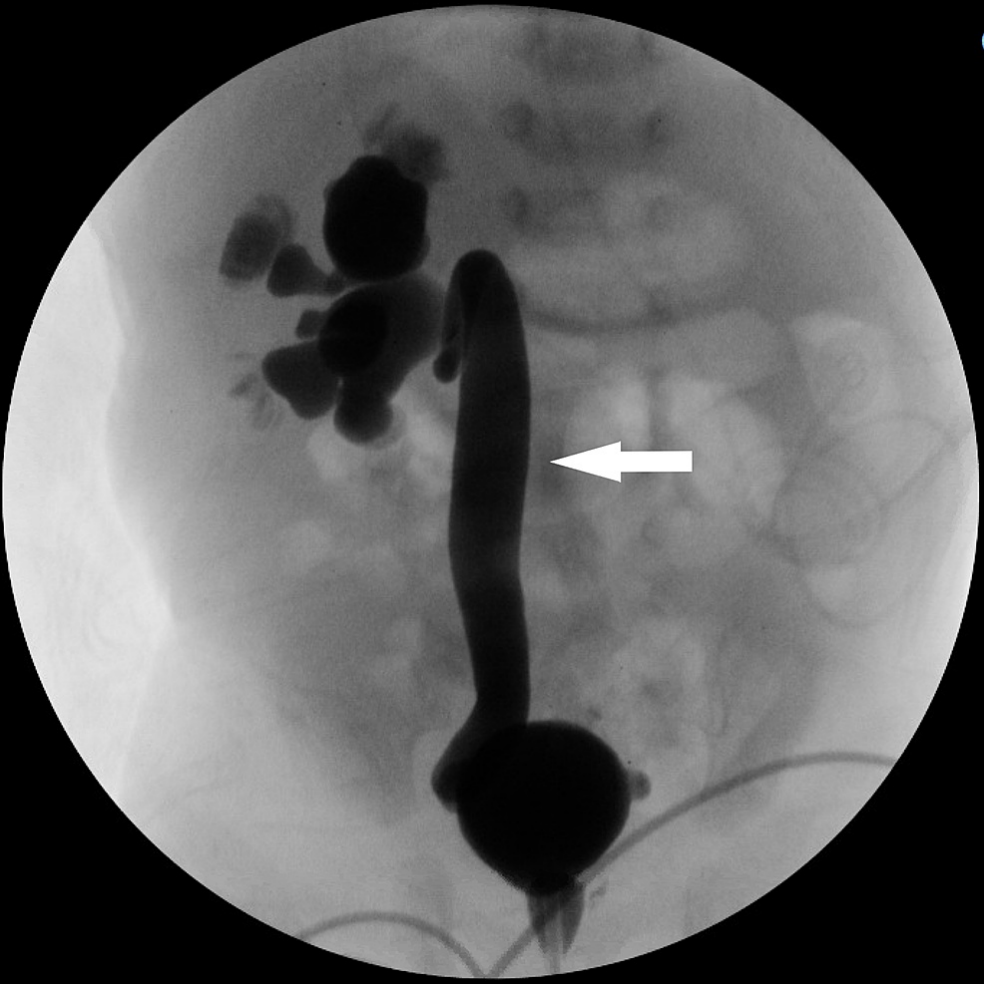
Voiding cystourethrogram revealing grade 5 right-sided vesicoureteral reflux

Because TAR syndrome patients often experience abnormalities within other organs and systems, further detailed workups were performed. The head ultrasound (US) to rule out bleeding was unremarkable. The echocardiogram was normal. The renal US revealed moderate right hydronephrosis (Figure 3). Voiding cystourethrogram (VCUG) showed grade 5 right-sided VUR (Figure 4). Nuclear medicine renogram revealed differential renal function: 48% for the right kidney and 52% for the left. Finally, the spinal US was normal.

After the VCUG, urology recommended initiating urinary tract infection (UTI) prophylaxis with nitrofurantoin. However, the patient became febrile and tachycardic on day of life (DOL) nine. A full septic workup, including blood, urine, and cerebrospinal fluid (CSF) cultures, was performed. The platelet count had decreased to 22,000/uL. Urosepsis was presumed, and empiric antibiotics were initiated with ampicillin and gentamicin. The patient required two platelet transfusions before lumbar puncture (LP) could be safely performed; thus, LP was performed 24 hours after initiation of empiric antibiotics. Blood and urine cultures were both positive for *Enterobacter aerogenes*. After antibiotics had been administered for 24 hours, the CSF findings were inconclusive: CSF culture was negative, CSF glucose was low, CSF protein was high, and CSF white blood cell (WBC) count was 9/uL. Antibiotics were changed to gentamicin and cefepime in view of the culture results and inconclusive CSF findings. The patient completed seven days of gentamicin and 14 days of cefepime.

The patient was discharged home on DOL 24 on Bactrim 2 mg/kg/day for UTI prophylaxis. The platelet count at discharge had improved to 96,000/uL. Two days before discharge, he was changed from Nutramigen to a trial of formula that included cow’s milk, Enfamil newborn. The patient did not show any signs or symptoms of CMPA while on cow's milk-based formula.

At six weeks of life, the patient presented to the emergency department with vomiting. Upon physical exam, he was found to have an erythematous diaper rash. Other findings included low platelet count (68,000/uL), normocytic anemia, and UTI. Urine culture was positive for *Escherichia coli*, which was resistant to Bactrim but susceptible to nitrofurantoin. The patient was admitted to the pediatric ward. His GI symptoms resolved after changing the formula back to Nutramigen, and he was diagnosed with CMPA. His normocytic anemia was hypothesized to be secondary to GI bleeding due to this allergy. His UTI was treated with nitrofurantoin, and he was discharged home after a three-day stay in the hospital on UTI prophylaxis with amoxicillin.

## Discussion

TAR syndrome is an inherited autosomal recessive condition. The diagnosis may be confirmed if a patient has a microdeletion on chromosome 1q21, resulting in a null allele of the RBM8A gene and a hypomorphic allele of the other RBM8A gene due to a pathogenic variant SNP [4]. In these patients, one copy of the RBM8A gene is non-functional due to the microdeletion and the other copy is not expressed normally due to the rare, non-coding SNP [1,9]. Diagnosis of the syndrome can occur prenatally by ultrasound and platelet count via fetal blood sampling via cordocentesis [10]. At birth, the absence of radii and the presence of thrombocytopenia, with or without other manifestations of TAR, are considered hallmarks of a positive diagnosis [6].

The thrombocytopenia that develops in TAR syndrome is HMT, platelet hypoproduction caused by insufficient numbers of megakaryocytes, the precursors to platelets, in the bone marrow [7]. The etiology of the HMT in these patients is hematopoietic stem cells, which do not appropriately respond to thrombopoietin and impaired maturation of the megakaryocyte progenitor cells in the bone marrow [7]. Thrombocytopenia may be present at birth among those with TAR syndrome, with 95% of those with the condition developing significant thrombocytopenia within four months of birth [4]. Platelet counts appear to increase with age and can appear normal in later childhood/adulthood [10]. Moreover, there is a possibility of exacerbation of the underlying thrombocytopenia by CMPA, as seen in our case. The mechanism of the exacerbation is reported to be direct immunoglobulin E (IgE) immune-mediated or secondary to increased GI bleeding due to loss of coagulation proteins [11]. It is important that healthcare professionals treating those with TAR are aware of its impact on platelet count and act accordingly when performing surgical procedures [7].

Patients with TAR syndrome have absent radii with preserved fingers and thumbs bilaterally [12]. These patients tend to have a contracture of the joint, which causes flexion and radial deviation of their hands, resulting in the hands often being at right angles to the forearms [13]. The syndrome frequently results in hypoplasia of the muscles and soft tissues in the shoulders and arms [13]. Shoulder abnormalities associated with TAR syndrome may include absent or hypoplastic scapula, glenoid fossa, clavicle, or acromion.

TAR syndrome has been known to be associated with multiple cardiac, gastrointestinal, renal, skeletal, and hematologic anomalies [14]. Although the rarity of TAR syndrome limits our ability to explore its epidemiology through the use of large datasets, one clinical genetic study explored the phenotypes of 34 patients with confirmed TAR syndrome [8]. They found all the cases had thrombocytopenia and bilateral radial aplasia; concurrently, 47% also had lower limb malformations, 47% had CMPA, 23% had renal anomalies, and 15% had cardiac anomalies [8].

Renal and genitourinary abnormalities may be associated with TAR syndrome. Case reports have described patients with TAR syndrome as having horseshoe kidneys [15], penoscrotal transposition [16], bilateral hypoplastic kidneys with decreased renal function [17], and crossed fused renal ectopia [18]. However, to our knowledge, VUR and hydronephrosis have not been previously reported in a patient with TAR syndrome.

The mortality of those with TAR syndrome is unknown; however, data from Hedberg and Lipton's review of 100 cases of TAR syndrome found 21 deaths out of 77 cases [19]. Of these 21 deaths, 14 (66%) occurred before the age of four months, six (28%) occurred between the ages of four and 14 months, and only one (5%) occurred after the age of 14 months [19]. The cause of death was only known in 18 cases; of these, 16 of 18 (89%) were due to hemorrhage, mostly intracranial, one (5.5%) was due to sepsis, and one (5.5%) due to congestive heart failure in a patient who also had tetralogy of Fallot [19]. Hedberg and Lipton found that most infants with TAR will survive past infancy with aggressive supportive therapy with platelet transfusions [19]. Patients with TAR have not been found to have intellectual disability except in the case of intracranial hemorrhage [19]. Despite the small sample size internationally, the current evidence is suggestive of the vast impact TAR syndrome can have upon the health of those diagnosed with it and demonstrates the key role healthcare professionals have in this regard.

## Conclusions

TAR syndrome is an exceedingly rare genetic condition with a clear presentation. It is associated with increased morbidity and mortality among children diagnosed with it. Potentially severe when diagnosed within the first few weeks of life, the condition can be effectively treated, and its consequences reduced.

Our aim in publishing the current case report is that healthcare professionals will be able to use this information to effectively treat those diagnosed with TAR in the future. The genetic basis of the syndrome, as well as its significant impact on health, calls for further research into the etiology of the condition, as well as how best to mitigate its consequences via effective treatment.

## References

[1] Tassano E, Gimelli S, Divizia MT, Lerone M, Vaccari C, Puliti A, Gimelli G (2015). Thrombocytopenia-absent radius (TAR) syndrome due to compound inheritance for a 1q21.1 microdeletion and a low-frequency noncoding RBM8A SNP: a new familial case. Mol Cytogenet.

[2] Hall JG, Levin J, Kuhn JP, Ottenheimer EJ, van Berkum KA, McKusick VA (1969). Thrombocytopenia with absent radius (TAR). Medicine (Baltimore).

[3] Kumar C, Sharma D, Pandita A, Bhalerao S (2015). Thrombocytopenia absent radius syndrome with tetralogy of Fallot: a rare association. Int Med Case Rep J.

[4] Cowan J, Parikh T, Waghela R, Mora R (2020). Thrombocytopenia with absent radii (TAR) syndrome without significant thrombocytopenia. Cureus.

[5] Toriello HV (2011). Thrombocytopenia-absent radius syndrome. Semin Thromb Hemost.

[6] Alagbe OA, Alagbe AE, Onifade EO, Bello TO (2019). Thrombocytopenia with absent radii (TAR) syndrome in a female neonate: a case report. Pan Afr Med J.

[7] da Costa DV, de Araújo VE, de Abreu FA, Souto GR (2020). Thrombocytopenia-absent radius (TAR): case report of dental implant and surgical treatment. J Clin Exp Dent.

[8] Greenhalgh KL, Howell RT, Bottani A (2002). Thrombocytopenia-absent radius syndrome: a clinical genetic study. J Med Genet.

[9] Albers CA, Newbury-Ecob R, Ouwehand WH, Ghevaert C (2013). New insights into the genetic basis of TAR (thrombocytopenia-absent radii) syndrome. Curr Opin Genet Dev.

[10] Bertoni NC, Pereira DC, Araujo Júnior E, Bussamra LC, Aldrighi JM (2016). Thrombocytopenia-absent radius syndrome: prenatal diagnosis of a rare syndrome. Radiol Bras.

[11] Mokha J, Serrano M (2013). Thrombocytopenia associated with cow's milk protein allergy: a case report. Clin Pediatr (Phila).

[12] Guastadisegni MC, Roberto R, L’Abbate A (2012). Thrombocytopenia-absent-radius syndrome in a child showing a larger 1q21.1 deletion than the one in his healthy mother, and a significant downregulation of the commonly deleted genes. Eur J Med Genet.

[13] Al Kaissi A, Girsch W, Kenis V (2015). Reconstruction of limb deformities in patients with thrombocytopenia-absent radius syndrome. Orthop Surg.

[14] Elmakky A, Stanghellini I, Landi A, Percesepe A (2015). Role of genetic factors in the pathogenesis of radial deficiencies in humans. Curr Genomics.

[15] Bradshaw A, Donnelly LF, Foreman JW (2000). Thrombocytopenia and absent radii (TAR) syndrome associated with horseshoe kidney. Pediatr Nephrol.

[16] Chappel BS (1958). Transposition of external genitalia in a case with Fanconi type deformity. J Urol.

[17] Fivush B, McGrath S, Zinkham W (1990). Thrombocytopenia absent radius syndrome associated with renal insufficiency. Clin Pediatr (Phila).

[18] Ahmad R (2007). A rare association of crossed fused renal ectopia. BMC Nephrol.

[19] Hedberg VA, Lipton JM (1988). Thrombocytopenia with absent radii. A review of 100 cases. Am J Pediatr Hematol Oncol.

